# Sequences Sufficient for Programming Imprinted Germline DNA Methylation Defined

**DOI:** 10.1371/journal.pone.0033024

**Published:** 2012-03-05

**Authors:** Yoon Jung Park, Herry Herman, Ying Gao, Anders M. Lindroth, Benjamin Y. Hu, Patrick J. Murphy, James R. Putnam, Paul D. Soloway

**Affiliations:** 1 Department of Nutritional Science and Food Management, Ewha Womans University, Seoul, Republic of Korea; 2 Division of Nutritional Sciences, College of Agriculture and Life Sciences, Cornell University, Ithaca, New York, United States of America; 3 Genetics and Development Graduate Field, Cornell University, Ithaca, New York, United States of America; Wellcome Trust Centre for Stem Cell Research, United Kingdom

## Abstract

Epigenetic marks are fundamental to normal development, but little is known about signals that dictate their placement. Insights have been provided by studies of imprinted loci in mammals, where monoallelic expression is epigenetically controlled. Imprinted expression is regulated by DNA methylation programmed during gametogenesis in a sex-specific manner and maintained after fertilization. At *Rasgrf1* in mouse, paternal-specific DNA methylation on a differential methylation domain (DMD) requires downstream tandem repeats. The DMD and repeats constitute a binary switch regulating paternal-specific expression. Here, we define sequences sufficient for imprinted methylation using two transgenic mouse lines: One carries the entire *Rasgrf1* cluster (RC); the second carries only the DMD and repeats (DR) from *Rasgrf1*. The RC transgene recapitulated all aspects of imprinting seen at the endogenous locus. DR underwent proper DNA methylation establishment in sperm and erasure in oocytes, indicating the DMD and repeats are sufficient to program imprinted DNA methylation in germlines. Both transgenes produce a DMD-spanning pit-RNA, previously shown to be necessary for imprinted DNA methylation at the endogenous locus. We show that when pit-RNA expression is controlled by the repeats, it regulates DNA methylation in *cis* only and not in *trans*. Interestingly, pedigree history dictated whether established DR methylation patterns were maintained after fertilization. When DR was paternally transmitted followed by maternal transmission, the unmethylated state that was properly established in the female germlines could not be maintained. This provides a model for transgenerational epigenetic inheritance in mice.

## Introduction

Proper programming of epigenetic states is essential for normal development. Many *trans* acting factors regulate these states, however, little is known about the *cis* acting signals that direct them to specific locations in the genome. We use the term programming to refer to any process by which appropriate epigenetic states are acquired. Imprinted genes have been useful models for characterizing *cis* acting epigenetic programming signals because of their predictable patterns of developmentally regulated programming. Imprinted epigenetic states from the previous generation are reset during primordial germ cell development between embryonic day 10.5 (e10.5) to e12.5 [1]; new epigenetic states are re-established in a sex-specific manner beginning at approximately e14.5 in the germline and after birth in males, and during oocyte growth in females [2], [3], [4]. Once established, imprinted methylation is maintained after fertilization throughout somatic development [2], [5]. Sex-specific epigenetic states are found at differentially methylated domains (DMD) or regions (DMR) within the imprinting control regions (ICR) of imprinted genes and act to enforce imprinted expression from one allele.

Studies have identified sequences within ICRs that control imprinting at *Igf2r*, *SNRPN*, *H19*, *Gtl2* and *Rasgrf1*. Large transgenes containing an intact *Igf2r* locus were able to recapitulate all features of imprinting at the locus, however, transgenes deleting the Region 2 DMR abolished normal imprinting, demonstrating Region 2 was necessary for imprinting control [6]. Additional transgenic studies implicated portions of Region 2 in controlling establishment of DNA methylation [7], however, the importance of these sequences at the endogenous *Igf2r* gene or throughout development is unknown. Sequences upstream of *SNRPN* commonly deleted in Prader-Willi and Angelman syndrome patients were implicated programming local maternal allele DNA methylation. When used in transgenic studies, those sequences were shown to be sufficient for programming DNA methylation [8], [9]. However, distal *SNRPN* sequences that are important for transcriptional control are also important for regulating methylation imprints in the female germline [10]. Transgenic mice bearing large portions of the human [11] and mouse [12] *H19* genes underwent normal establishment of DNA methylation in the germline of mice, though, methylation was not maintained during somatic development on the human transgene. It is not clear what are the positive signals that attract DNA methylation to the paternal copy of *H19*, however, they are likely to reside on a 15.7 kbp region [13]. Furthermore, CTCF and its binding sites are needed to exclude DNA methylation from the maternal allele [14], [15], [16], [17]. Similarly, a sequence was identified at *Gtl2/Dlk* that excludes DNA methylation from the maternal allele [18]. *H19* DNA methylation acquired in the germline expands during somatic development and sequences needed for this expansion have been identified in mice [19], [20], [21]. At *Rasgrf1*, a repeated sequence element was identified that is necessary for establishing methylation at the adjacent DMD in the male germline [22]. This sequence is also necessary between fertilization and implantation for methylation to be maintained, but is dispensable after implantation [23]. DNA methylation at the DMD is essential for *Rasgrf1* expression in neonatal brain [22], [23], [24]. The *Rasgrf1* repeat is a promoter for a DMD spanning transcript, which is targeted for processing into piRNAs and referred to as a pit-RNA (piRNA targeted-RNA. Its transcription and processing is needed for DNA methylation at *Rasgrf1* [25].

Here, we extend our previous studies at *Rasgrf1*, identifying sequences that are sufficient for programming DNA methylation at the locus. Our results show that transgenes carrying the repeats and sites of piRNA similarity are sufficient for proper programming of DNA methylation at the DMD in the germline of male and female mice. In one of our transgenic models, the unmethylated state established in the female germline was not maintained in their progeny if the maternally transgene had any history of prior transmission through the male germline. This provides a model for transgenerational inheritance of aberrant epigenetic states.

## Materials and Methods

### Generation of transgenic mice

All animal work was conducted according to relevant national and international guidelines and, in compliance with these rules, was approved under protocol number 2002-0075 by the Institutional Animal Care and Use Committee at Cornell University. To prepare the RC transgene, the full-length 241,789 bp BAC clone, RP23-118J13 (GenBank: AC102545.9), was modified by recombineering to flank the repeat element with loxP sites, then microinjected into mouse embryos. Briefly, plasmid pYJC6 [23] containing the *Rasgrf1* DMD, loxP-flanked repeats, and a frt-flanked *neo* cassette driven by the mouse *Pgk* promoter, was modified to have a dual promoter (*em7* and *Pgk* promoter) to produce pYP1. pYP1 was recombined into the BAC by homologous recombination in SW105 cells. After BAC recombineering, the *neo* cassette was excised by flpe recombination, resulting in the BAC RC. RC BAC DNA was prepared using the Nucleobond BAC Maxi kit (Clontech, 635941) according to manufacturer's instructions and resuspended in 0.1× TE (1 mM Tris-HCl, 0.1 mM EDTA; pH7.5) prior to microinjection. To prepare the DR transgene, the *Eco*RI site 5′ of DMD and *Eco*RV site 3′ of the floxed repeats in plasmid pYJC6 were modified to *Asc*I sites and the *Asc*I fragment was then subcloned into *Asc*I sites of plasmid pNI [26] between the mouse gamma-globin enhancer and *neo* selectable marker to generate the plasmid pNIDR4. pNIDR4 was linearized with *Apa*LI to remove the plasmid backbone, digested DNA was gel purified with QiaEXII kit according to manufacturer's instructions and resuspended in 1× injection buffer (10 mM Tris-HCl, 0.1 mM EDTA; pH7.5). Pronuclear injections for both constructs were performed in FVB/N embryos. Founder animals were bred to wild type FVB/N mice to establish independent transgenic lines and all crosses using transgenic mice were done with animals on that inbred background.

### DNA isolation and restriction enzyme assays for methylation

Somatic DNA was prepared by SDS lysis buffer as previously described [27]. For oocyte DNA, 10 to 30 oocytes were collected from infundibula of two to four independent superovulated mice. DNA was prepared as described above with the added difference that glycoblue was added to enhance DNA precipitation and visualization. Sperm DNAs were collected from >6week old mice by allowing sperm to swim out from the caudal epididymi in PBS. Cells were resuspended in GTC solution (5.4 M guanidine thiocyanate, 1.5% sarcosyl, 100 mM Tris pH8.0, 200 mM β-mercaptoethanol) and incubated at 42°C for 30 min, followed by proteinase K digestion at 55°C overnight. All DNAs were recovered by isopropanol precipitation. Southern blots used to detect RC and DR DMD methylation were probed with BRP1.0 [22] and a globin enhancer probe, respectively. Methylation-sensitive PCRs used *Hha*I digested DNAs followed by PCR with primers used for genotyping (Table 1).

**Table 1:** Primers for genotyping.

Name	Sequence	Lab Code	Use
RC_LoxP	F: CTGCACCGCTGCCGCTAAGC	23	Specific for 5′ of loxP flanked repeats in RC
	R: CCTGCAGGTCGACATAACTTC	24	
RC_Rep	F: TTTCTGCCATCATCCCAGCC	18	280 bp in RC due to loxP and Frt sites, 190 bp in endogenous allele
	R: TGTCCTCCACCCCTCCACC	17	
RC_ e14	F: ATGATTGAACAAGATGGATTGCAC	249	Expression analysis of *Rasgrf1* exon14
	R: ATTGGTGAAGACGCGATAGG	250	
RC_A19p	F: CGCAGTTCCAATAAGCATCA	75	Within A19 promoter, RC carries a *Bcc*I site
	R: CTGGTTTGCCATCAGGAAAT	76	
DR_ LoxP	F: CTGCACCGCTGCCGCTAAGC	23	Specific for 3′ of loxP flanked repeats in DR
	R: CCTGCAGGTCGACATAACTTC	24	
DR_ Neo	F: ATGATTGAACAGATGGATTGCAC	52	Specific for *neo* cassette
	R: TTCGTCCAGATCATCCTGATCGAC	53	

### Bisulfite sequencing and COBRA

Somatic and sperm DNAs (1 µg) from RC and DR mice were digested with *Hind*III/*Pvu*II and *Pvu*II, respectively, purified by phenol/chloroform extraction, resuspended in 20 µl, denatured at 95°C for 5 min, then incubated with 1 µl of 6.3 M NaOH at 39°C for 30 min. The samples were mixed with 208 µl bisulfite solution (3.9 M Na Bisulfite pH 5.1, 0.66 mM hydroquinone) inclubated at 55°C for 16 hr, desalted, incubated with 2.5 µl 6.3 M NaOH 37°C for 15 min, and desalted again. PCR amplification was for 42 cycles with unbiased primers listed in Table 2. Oocyte DNA samples, contained less than 1 µg DNA and were amplified by two rounds of PCRs using 30 and 40 cycles. PCR products were cloned by TOPO pCR2.1 kit (Invitrogen) and the clones, at least 10 per each PCR, were sequenced. All bisulfite data shown were from two to three mice per assay. For COBRA, PCR products were digested with *Bst*UI.

**Table 2:** Primers for Bisulfite PCR.

Name	Sequence	Lab Code	Use
RC_BS	F: AGAGAGTATGTAAAGTTAGAGTTGTGTTGTTG	225	For all RC DNAs
	R: ATAAACTACTACAACAACTT	414	
DR_BS	F: AATAGGGTATTAGTGAAAGTTTGTAATGAATT	364	For DR sperm DNA
	R: CAAAAACAACAATAATAACAAAAACAAAAACAATAT	272	
DRbt_BS	F: TAGTAGTAGTGGTTGGGGTAGGGGTAGT	638	For DR somatic & oocyte DNA
	R: ACAAAATACCAATAAAAATCTACAATAAATTC	641	

### Gene expression analysis

To distinguish *Rasgrf1* expression from RC transgenic and endogenous alleles, RT-PCR was done using poly dT primed cDNA made from neonatal brain (post partum day 2) and the following primers: Forward in exon 13: 5′-GGCTCATGATGAATGCCTTT-3′, Reverse in exon 15: 5′-TACAGAAGCTTGGCGTTGTG-3′ annealed at 58°C with 40 cycles of amplification. The PCR product was digested with 10 U *Aci*I. To monitor pit-RNA expression, we used a common forward primer for the transgenic and endogenous pit-RNA: 5′-CTGCACCGCTGCCGCTAAGC-3′; and one of two reverse primers: 5′-ATCACTAGTGCGGCCGGCCGCCTGCA-3′, which is transgene specific, or 5′-GCAGCAGTAGCAGTCGTGGT-3′ which does not distinguish transgenic from endogenous pit-RNAs. Annealing was at 62°C with 36 cycles of amplification.

## Results

### Generation and characterization of transgenic mice

**Figure 1:**
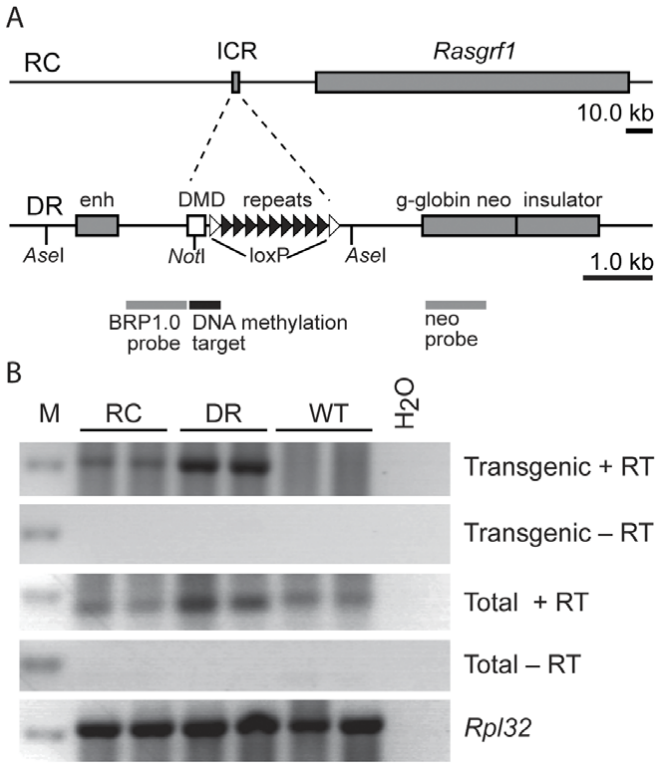
Constructs used to generate transgenic mice and their DMD transcripts. A, Top, the RC transgene was generated from BAC clone RP23-118J13, using recombineering to flank the repeats with loxP-sites. It carries 110 kb upstream and 10 kb downstream genomic sequences containing the entire *Rasgrf1* gene. A, Bottom, the DR transgene contains only DMD and loxP flanked repeats from the *Rasgrf1* locus in a vector that carries a mouse beta-globin enhancer, the neo cassette, and a the chicken HS4 insulator [26]. Region for all DNA methylation analysis, by bisulfite and restriction methods are shown, as well as probes for Southern blots. B, RT-PCR analysis of DMD spanning pit-RNA transcripts. RNAs made from adult testes of two independent RC, DR and wild type mice (WT) were assayed using primers specific for both transgenes (Transgenic) or that did not distinguish between endogenous and transgenic transcripts (Total). PCR reactions were done with (+RT) or without (−RT) reverse transcription. *Rpl32* was used as a control.

The RC transgene was prepared using the full-length 242 kbp BAC clone containing C57BL/6 genomic sequences extending from 110 kb upstream from *Rasgrf1* to 10 kb downstream of the gene (Fig. 1A, top). The BAC contains the DMD, repeats and the entire *Rasgrf1* coding region. A second transgene (DR) was prepared that carries the DMD and repeats, but no other sequences from *Rasgrf1* (Fig. 1A, bottom). Both transgenes have loxP sites on either side of the repeats, confirmed not to affect normal *Rasgrf1* imprinting at the endogenous locus [23], but allowing us to distinguish the endogenous DMD from the DMD of the transgene. Transgenes were injected into FVB/N zygotes. Three founders in total were analyzed including one male RC founder, one male DR founder, and one female DR founder, each with three to five copies of the transgene. Mice were maintained on the FVB/N background as hemizygotes. The RC and DR transgenes supported expression of a DMD-spanning pit-RNA transcript in testes (Fig. 1B), which we previously showed is transcribed from the repeats in wild type mice and is processed into piRNAs [25]. Factors involved in piRNA binding and metabolism are required for imprinted *Rasgrf1* methylation [25].

### RC transgene recapitulates imprinted expression of the endogenous gene

**Figure 2:**
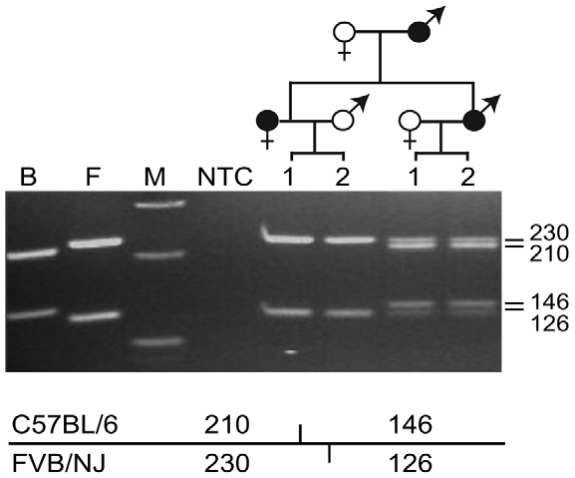
*Rasgrf1* expression is imprinted in neonatal brains of RC transgenic mice. Mice bearing the RC transgene were bred according the pedigree shown above the gel. Filled ♀ or ♂ symbols depict transgene positive mice, whereas unfilled symbols denote their non-transgenic mates. Progeny labeled 1 and 2 carry the transgene. To assay *Rasgrf1* expression from the FVB/N or C57BL/6 alleles, we digested a 356 nt RT-PCR amplicon with *Aci*I, which produced the distinct fragment sizes from the two alleles as shown below the gel. The endogenous FVB/N allele was expressed in all mice, whereas the C57BL/6 transgenic RC allele was expressed only upon paternal transmission. Inbred FVB/N (F) and C57BL/6 (B) were used as controls. M, marker; NTC, no template control.

To determine if the BAC carried sequences necessary for imprinted *Rasgrf1* expression, RT-PCR was performed using RNA from brains of neonates with maternally or paternally transmitted RC transgenes. Neonatal brain is where *Rasgrf1* shows imprinted expression. C57BL/6 and FVB/N have SNPs in the RT-PCR amplicon that spans exon 13 to 15, allowing us to distinguish *Rasgrf1* expression from the endogenous FVB/N and C57BL/6 transgenic alleles, after *Aci*I digestion of the RT-PCR product. Analysis of neonatal brain showed the RC transgene expressed *Rasgrf1* only when transmitted through male germline, and was silenced upon maternal transmission (Fig. 2). Quantitative RT-PCR showed that mice with a paternally inherited RC transgene had 3.5 fold more *Rasgrf1* expression relative to mice with a maternally inherited RC transgene, which is proportional to our estimate that there are three to five copies of the integrated transgene (data not shown). This demonstrates the RC transgenic line recapitulates the imprinting pattern and the level of expression per copy, of the endogenous locus, and that all regulatory elements necessary for proper imprinted expression are contained on the 242 kb transgene.

### RC transgene recapitulates imprinted methylation and gametic reprogramming of the endogenous gene

**Figure 3:**
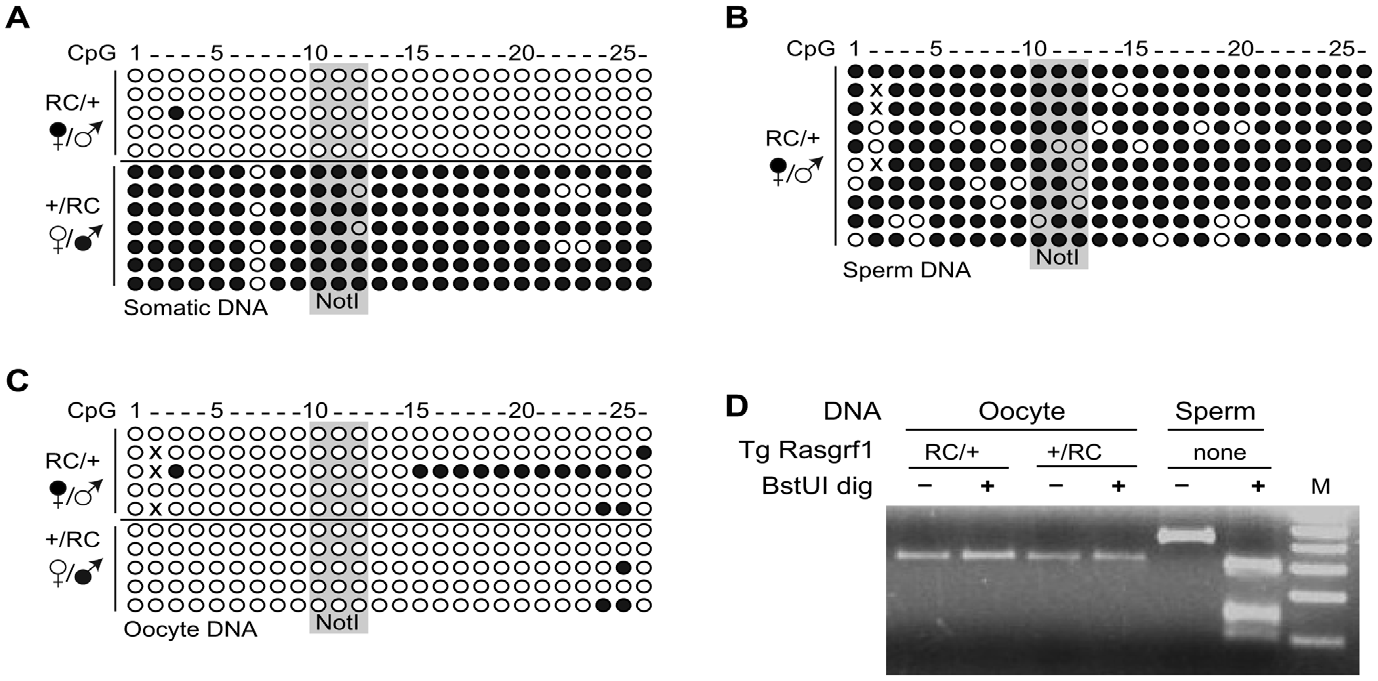
Imprinted DNA methylation and reprogramming in RC transgenic mice. Bisulfite methylation analysis of the transgenic DMD, querying 26 CG dinucleotides including three CpGs residing in a *Not*I site. Mice inherited the transgene from their mothers (RC/+) or fathers (+/RC). We analyzed somatic tail DNA (A), sperm DNA (B) and oocytes DNA (C). Open and filled circles denote unmethylated and methylated CpGs respectively; X denotes C nucleotides that were mutated during amplification to non C or non T residues and whose methylation state could not be inferred. Insertion of LoxP in the transgene enabled transgene specific analysis. D. COBRA analysis of methylation state in oocyte DNAs entailed amplifying bisulfite treated DNAs by primers specific for the transgene, then digesting the PCR products with *Bst*UI, which cut at its CGCG site only if the genomic DNA was methylated. As a control, the assay was done with sperm DNA from wild type mice and primers for the endogenous allele, which produced a slightly different sized product. M, marker.

Next, we determined the DNA methylation status of the RC transgene in somatic and germline DNAs from animals inheriting the transgene maternally or paternally. When we used bisulfite analysis to assay transgene methylation in tail DNAs of mice inheriting the transgene from their fathers, we observed hypermethylation of the transgenic DMD, whereas after maternal transmission, the DMD was hypomethylated (Fig. 3A). The results were confirmed by Southern blots (data not shown). This indicated that the transgene contained all elements to impose imprinted DNA methylation in somatic tissue, as well as imprinted *Rasgrf1* expression.

At the endogenous locus, paternal-specific methylation is established during spermatogenesis and is erased during oogenesis. To determine whether the reprogramming occurred appropriately on the RC transgenes, we used bisulfite analysis to assess DNA methylation in sperm and oocytes. Methylation at the transgenic DMD recapitulated the patterns seen at the endogenous locus: They were methylated in sperm (Fig. 3B) and unmethylated in mature oocytes (Fig. 3C). The results from sperm were confirmed by Southern blot and COBRA analysis (data not shown) and results from oocytes were confirmed by COBRA analysis (Fig. 3D), demonstrating the RC transgene was properly reprogrammed during gametogenesis in a sex-specific manner. Collectively, our analyses show that the RC transgene recapitulates all aspects of *Rasgrf1* imprinting.

The DMD-spanning pit-RNA, which is a key regulator of *Rasgrf1* DNA methylation in the male germline, is transcribed from the *Rasgrf1* repeats [25] and is made by the RC transgene (Fig. 1B). We asked if repeat-derived pit-RNA transcripts can work in *trans* to effect methylation, or if their action is restricted in *cis.* To make this determination, we generated mice that carried a paternally inherited copy of RC and homozygous deletions of the endogenous *Rasgrf1* repeats. In these mice, the RC transgene was the only source of the pit-RNA in the male germline. When we assayed DNA methylation in sperm of these mice, or in somatic DNA of their progeny with identical genotypes, we found that DNA methylation was present only on the transgene and not on the endogenous allele (Fig. S1). This demonstrated that pit-RNA transcribed from the repeats functions only in *cis* and not in *trans*.

### Male gametes establish imprinted methylation on the DMD of DR transgene, as seen at the endogenous *Rasgrf1* DMD

Having shown that the RC transgene carries all sequences necessary for *Rasgrf1* imprinting, we sought to define the sequences that are sufficient for this programming. Because previous studies showed the repeat sequences adjacent to the DMD were necessary for programming events, we asked if they were, by themselves, sufficient. The DR transgene, which contained the DMD and loxP-flanked repeats from *Rasgrf1* (Fig. 1, bottom), allowed us to address this question. The vector was a modified version of a previously described plasmid [26] we used to test enhancer blocking activity of *Rasgrf1* ICR sequences [28]. From our microinjections, we worked with one female (mouse 3047) and one male founder (mouse 2771).

**Figure 4:**
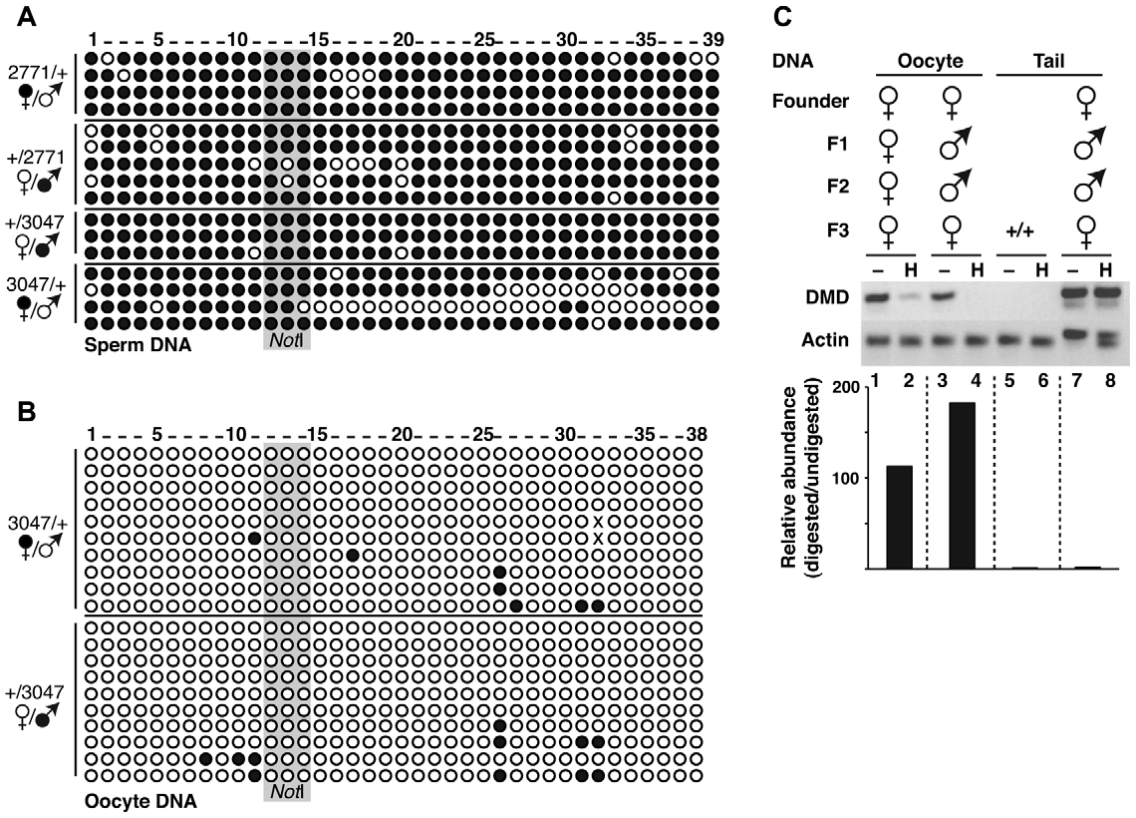
Sufficiency of the DR transgene for establishing and reprogramming imprinted DNA methylation during gametogenesis. Bisulfite analysis of DNA from sperm (A) or oocytes (B) of animals inheriting the DR transgene. Mice derived from male founder 2771 inherited the transgene from their mothers (2771/+) or fathers (+/2771); mice derived from female founder 3047 inherited the transgene from their mothers (3047/+) or fathers (+/3047). Transgene-bearing parent is indicated with a filled ♀ or ♂ symbol. Results are presented as in Fig. 3. Note that additional CpGs not in the endogenous DMD that were present on the transgene were also reprogrammed. C. DNA methylation in transgenic mice descended from a three generation pedigree from female founder 3047. Abbreviated pedigree (top) shows only transgene-carrying mice and the sex of the animal transmitting the transgene at each generation. Oocyte or tail DNAs from F3 transgenic mice were amplified before (−) or after (H) *Hha*I digestion. Tail DNA from wild type (+/+) was used as a negative control. The PCR was done using primers shown in Table 2 specific for the DMD of the transgene, which span five *Hha*I sites, or primers from actin, which span no *Hha*I sites. Gels (middle) or Q-PCR results (bottom) are shown with relative abundance of the signals from cut *vs.* uncut DMD amplifications indicated.

To test whether the DR transgene carries methylation-programming elements sufficient to recapitulate imprinted methylation, we performed crosses to pass it through the male and female germlines. We then analyzed DNA methylation in the gametes of mice transmitting the transgene to assess whether germline DNA methylation had been programmed on the transgene as it is at the endogenous DMD. We also isolated somatic DNA from their progeny to determine if any previously established DNA methylation patterns were properly maintained after transmission. If the DR transgene carried sequences sufficient for imprinted DNA methylation programming, we expected that the methylation programming sequences on the transgene would direct methylation to the transgenic DMD in sperm DNA, regardless of whether or not the males from which sperm were taken inherited the transgene from their mothers or fathers. To test this, we derived mice from crosses using the male founder, which involved simply breeding him with a non-transgenic female. For crosses using the female founder, this involved breeding her with a non-transgenic male and then breeding her transgene-carrying sons with a non-transgenic female. To assess the methylation state of the transgenic DMD in sperm, we performed bisulfite analysis of 24 CpGs within the transgenic DMD that are shared with the endogenous DMD and 15 additional CpGs that are transgene specific. The endogenous *Rasgrf1* DMD acquires DNA methylation at each of 26 CpGs in the male germline, and this is erased in the female germline [22]. The analysis showed that regardless of the founder pedigree or the mode of inheritance, the transgenic DMDs were hypermethylated in sperm DNA of eight independent males tested (Fig. 4A). This demonstrated that the transgene carried the *Rasgrf1* sequences that were sufficient for establishing properly programmed DNA methylation of the transgenic DMD in the male germline.

### Female gametes establish the unmethylated state on the DMD of DR transgene, as seen at the endogenous *Rasgrf1* DMD

We next wanted to know if the transgene could assume the unmethylated state upon passage through the female germline. Because preexisting DNA methylation at imprinted loci is normally erased in primordial germ cells, and because the *Rasgrf1* methylation promoting sequences do not induce DNA methylation in the female germline, we expected that maternal transmission of the transgenes would lead to loss of DNA methylation in oocytes. Furthermore, as was true for males, we expected to see this lack of methylation in oocytes regardless of whether or not the females from which they were taken inherited the transgene from their mother or father. To determine if the oocytes properly programmed the transgene to the unmethylated state, we isolated oocytes from transgene-bearing females, pooled them, prepared DNA and performed bisulfite sequencing of oocytes DNAs using transgene specific primers (Fig. 4B). In addition, we tested oocytes for methylation by amplifying the transgenic DMD before or after digestion with *Hha*I (Fig. 4C). If the transgenic DMD was unmethylated in oocytes, digestion will interfere with amplification. Results from both assays consistently showed that oocytes of all transgenic mice tested were unmethylated on the transgenic DMD.

These data demonstrated that the transgene underwent proper establishment of imprinted DNA methylation patterns that occur at the endogenous *Rasgrf1* locus in the germlines of male and female mice. Therefore, the 400 bp DMD and the 2 kbp repeat sequences, which are necessary for faithfully programming germline imprinting at *Rasgrf1* are also sufficient for this activity. The primers we used to assay the methylation state of the DR transgene detected an additional 14 CpGs that are not present on the endogenous DMD and transgene specific. Because these also were reprogrammed identically to the CpGs carried on the DMD, the repeat sequences are sufficient to impart imprinted DNA methylation to adjacent foreign sequences.

### Imprinted methylation established on the transgenic DMD in male gametes is maintained during somatic development

**Figure 5:**
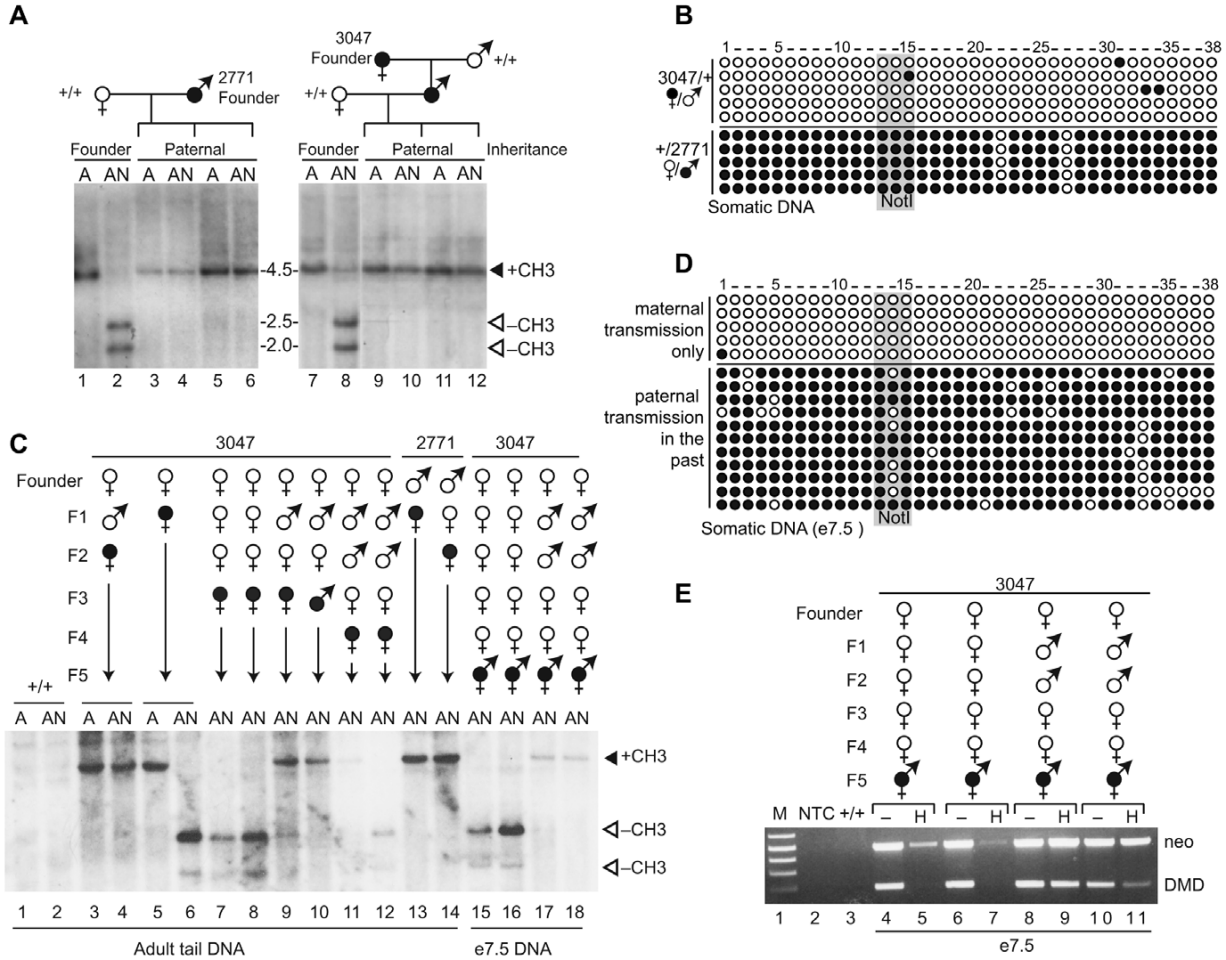
Transgenerational inheritance of DNA methylation states on DR transgenes. A. Methylation states at DR transgenes in somatic tissue after paternal transmission were assayed by methylation-sensitive Southern blots. Pedigrees were derived from male founder 2771 (left) and female founder 3047 (right). Transgene-bearing parents in the pedigrees are indicated by filled ♀ or ♂ symbols. DNAs from transgene bearing progeny (lanes 3–6 and 9–12) as well as from the founders themselves (lanes 1–2 and 7–8) were digested with *Ase*I alone (A) or in combination with *Not*I (AN). Persistence of the 4.5 kb band after *Ase*I and *Not*I double digestion indicates the transgene is methylated. Appearance of the 2.5 and 2.0 kb bands indicates the transgene is unmethylated. B. Bisulfte analysis of tail DNA from mice in A bearing a maternally transmitted transgene from female founder 3047 or a paternally transmitted transgene from male founder 2771. C. Transgenerational inheritence of DNA methylation states. Multiple generation pedigrees were developed from female founder 3047 and male founder 2771 (top). For simplicity, full pedigrees not diagrammed. Transgene-bearing descendents of the founders are shown by generation (F1 through F5) along with the sex of each transgene-bearing mouse used to prepare the pedigree. DNAs were prepared from transgenic animals with filled ♀ or ♂ symbols and used for methylation analysis by Southern blot as in A. DNAs came from adult tails (lanes 1–14) and day 7.5 embryos (lanes 15–18). Control DNAs were included from wild type mice (+/+ lanes 1–2), which show no hybridization to the transgene-specific probe, and from mice inheriting their transgene directly from their fathers (lanes 3–4, 13), which led to transgene methylation. D. Bisulfte analysis of DNA from embryonic day 7.5 samples used in lane 15–18 in C. E. Methylation sensitive PCRs using *Hha*I digested DNA from e7.5 samples used in C (lane 15–18) and in D. PCR amplification after *Hha*I digestion indicated DMDs or neo cassettes on DR transgenes were methylated. M, size marker; NTC, no template control; +/+, wild type control.

We next wanted to determine if the *Rasgrf1* repeat sequences are also sufficient for maintaining, in somatic tissue, the imprinted DNA methylation patterns they established in the germlines. For this test, we performed crosses to pass the DR transgene from both founders through the male germline, and then evaluated transgene DNA methylation in somatic DNA of the progeny. Similar to crosses using the male founder, this involved breeding him with a non-transgenic female, whereas for crosses using the female founder, this involved breeding her with a non-transgenic male and then breeding her transgene-carrying sons with a non-transgenic female. We isolated tail DNA from the progeny of transgenic sires derived from both founders and did Southern blots using the methylation-sensitive enzyme, *Not*I, and also bisulfite analysis. It is worth noting that the transgene in the male founder was unmethylated, as expected, because the transgene was integrated into his genome after microinjection, bypassing the male germline (Fig. 5A lanes 1–2). However, when the male founder passed the transgene through his germline to his progeny, it acquired methylation in their somatic tail DNA (Fig. 5A lanes 3–6). The bisulfite sequencing results were in complete agreement with the Southern blot data (Fig. 5B, bottom). The same somatic methylation patterns appeared when the male progeny of the female founder passed the transgene to the next generation (Fig. 5A lanes 9–12). These results demonstrated that the methylation programming sequences on the transgene faithfully recapitulated both establishment and maintenance of DNA methylation at ectopic sites that it performs at the endogenous *Rasgrf1* locus after passage through the male germline.

### Reprogrammed methylation state on the transgenic DMD in female gametes is not maintained during somatic development when the transgene was paternally transmitted in the past

The last test we performed was to determine if the unmethylated state that was programmed in oocytes could be maintained during somatic development in the next generation. As was true for the male founder, the female founder lacked DNA methylation in her somatic DNA (Fig 5A, lanes 7–8). We used her to establish two pedigrees. In one pedigree, we maintained maternal-only transmission of the transgene. In all transgenic progeny from this pedigree, the unmethylated state of the transgene was preserved (Fig. 5B, top and Fig. 5C, lanes 5–8, 15–16). In the other pedigree, we passed the transgene through male descendents of the female founder, and then through the female germline again. As we demonstrated in Fig 4B, the unmethylated state was properly re-established in the germlines of females derived from this second pedigree. However, intriguingly, the unmethylated state was not maintained during somatic development (Fig. 5C, lanes 9–11, 17–18). This was also true for mice derived from the male DR founder; the transgene acquired methylation in somatic DNA from progeny of his daughters (Fig. 5C lane 14). We observed only one exception to this failure to maintain the unmethylated state of a maternally transmitted transgene in pedigrees with past paternal transmission (Fig. 5C, lane 12). We have not determined the number of generations for which this aberrant state persists; however, epigenetic marks acquired in the male germline at the F2 generation persisted in somatic tissue of F5 mice, despite passage through the female germline in the F3 and F4 generations (Fig 5C, lanes 17 and 18). This transgenerational memory of the male epigenetic state was seen in descendents of both founders, indicating the failure to maintain the maternal transgenic DMD in the unmethylated state was not founder specific. The aberrant maternal allele methylation was acquired by embryonic day 7.5 (Fig. 5D and 5E) indicating that maintenance failure occurred between fertilization and that stage of development. The difference in methylation phenotypes between mice that had no past paternal transmission and those that did was highly significant (P<0.003; one-tailed Fisher's exact test). Collectively, our data from two transgenic founders clearly show that the mode of transgene transmission from previous generations influenced the DNA methylation in subsequent generations. This property is our operational definition of transgenerational epigenetic inheritance, few examples of which exist in experimental systems [29], [30], [31].

Our data show that although the transgene clearly carried all the sequences that are necessary and sufficient for establishing correct imprinted DNA methylation patterns in the male and female germlines, and also for maintaining paternal allele methylation, they were not sufficient to properly maintain the unmethylated maternal state in somatic tissue. One possible explanation is that additional sequences needed to maintain the unmethylated state on the maternal allele were absent from our transgene. It is also possible that additional sequences within the transgene, such as the CpG-rich, phage-derived *neo* cassette interfered with maintenance mechanisms, perhaps by attracting methylation that spread into the transgenic DMD. To explore this latter possibility, we assessed *neo* cassette DNA methylation in mice derived from the female founder, including animals in which the maternal allele was methylated or unmethylated. We observed weak *neo* methylation even when there was no maternal DMD methylation (Fig. 5E, lanes 5,7), and stronger *neo* methylation when DMD methylation was apparent (Fig. 5E lanes 9,11). This is consistent with the possibility that the *neo* cassette contributed to methylation of the maternal DMD and interfered with maternal maintenance mechanisms. The *neo* cassette and other features on the DR transgene were included to report whether imprinted DNA methylation patterns facilitated imprinted expression of *neo*; however, *neo* methylation prevented its expression after any mode of inheritance and could not be used to report imprinted expression.

Despite the possible influence of the *neo* cassette on acquisition of somatic methylation of the maternal allele, we conclude that the *Rasgrf1* repeat element is both necessary and sufficient to properly program establishment of imprinted DNA methylation at the adjacent DMD in the germline of mice. It actively places DNA methylation in the male germline, and allows erasure of methylation in the female germline. By this critical test of methylation reprogramming in the mouse germline, the DMD and repeats inserted at ectopic sites behave exactly as they do at the endogenous locus.

## Discussion

Previous work showed the tandem repeat elements downstream of the DMD were necessary for imprinted DNA methylation at the *Rasgrf1* locus [22]. Data described here show they are sufficient to recapitulate establishment of imprinted DNA methylation during gametogenesis at the DMD, directing DNA methylation in sperm and allowing erasure of DNA methylation in oocytes.

The RC transgene established proper patterns of imprinted DNA methylation in both the male and female germlines and also maintained those patterns after fertilization. Additionally, it exhibited proper patterns of imprinted *Rasgrf1* expression, and the level of expression was similar to the endogenous allele in proportion to the copy number. By all assays, the RC transgene behaved exactly like the endogenous locus. This indicated that all *cis*-elements required for *Rasgrf1* imprinting were included on the RC transgene.

The DR transgene, which carried only two sequence features from the *Rasgrf1* locus – the DMD and repeats – were by themselves sufficient for germline reprogramming of imprinted DNA methylation at ectopic sites. Three lines of evidence demonstrated this. First, progeny of the founders showed that the transgene had appropriate DNA methylation after transmission from the male founder, and absence of methylation when transmitted by the female founder. Second, in both independent lines, the established DNA methylation was properly erased in oocytes and new imprinted DNA methylation was established during spermatogenesis, indicating the repeats and DMD were sufficient to reprogram germline DNA methylation at ectopic locations in a sex-specific manner. Third, the transgene DNA methylation that was properly established during spermatogenesis was successfully maintained during somatic development. The unmethylated state properly established in oocytes was maintained during somatic development, however, only when there was no history of transmission through the male germline. Any history of paternal transmission of the transgene prevented faithful maintenance of the unmethylated state. Failure was seen as early as embryonic day 7.5, with only one exception, and epigenetic aberrations that arose in the F2 generation persisted into the F5 generation. Other reports described transmission of epigenetic states through the maternal or paternal germlines in experimental systems [29], [30] and humans [32], [33] after maternal or paternal transmission. In combination with our data, it is clear that epigenetic memories can be imposed by transmission through either germline.

The mechanisms underlying transgenerational epigenetic inheritance in our system are unknown. One likely mechanism involves antagonism between DNA methylation and H3K27me3, which controls epigenetic states at the endogenous *Rasgrf1* locus. DNA methylation on the paternal allele antagonizes placement of H3K27me3, and H3K27me3 on the maternal allele antagonizes placement of DNA methylation [34]. Both marks are sensitive to the *Rasgrf1* repeats. It is possible that the DR transgene has diminished capacity to recruit H3K27me3 on the maternal DMD after past paternal transmission, perhaps because low levels of DNA methylation acquired on the nearby *neo* sequences increases after past paternal transmission (Fig 5E). Consistent with this possibility is the observation that a transgene carrying a *beta-geo* cassette inserted in the 3′ UTR of beta-actin allele interfered with the maintenance of unmethylated CpG islands located in the beta-actin promoter [35].

Recent results identified several features of the mechanism by which the *Rasgrf1* repeats control DNA methylation [25]. First, the repeats constitute a promoter for a DMD spanning pit-RNA transcript that is expressed in embryonic testes. Second, the DMD spanning transcript contains at least two sites of complementarity to primary piRNAs and is targeted for processing into secondary piRNAs. Third, the piRNA pathway is needed for *Rasgrf1* methylation. Consistent with this mechanism is the fact that the RC and DR transgenes expresses a DMD spanning RNA. Interestingly, the DR transgene lacks sites of piRNA complementary found at the endogenouse locus, however, the RNA from DR carries additional sequences with identity to a known piRNA. We do not know how the pit-RNA triggers DNA methylation on the DMD; however, results with the DR transgene suggest that non-native sites of piRNA similarity are sufficient for local methylation.

In conclusion, RC transgene faithfully recapitulated *Rasgrf1* imprinting at ectopic chromosomal contexts, indicating that all the regulatory elements necessary and sufficient for imprinted methylation and transcription are included within the BAC used to prepare the transgene. The repeats and DMD on DR transgenes are two key features sufficient for establishing and reprogramming appropriate sex-specific DNA methylation in the male and female gametes. Studies are ongoing to characterize the pit-RNA requirements to support this epigenetic regulation.

## Supporting Information

Figure S1 **pit-RNA transcribed by the** ***Rasgrf1*** **repeats controls DNA methylation only in** ***cis*** **and not in** ***trans*** **.** A. Mice homozygous for a deletion of the *Rasgrf1* repeats (−/−) carrying a paternally inherited RC transgene (+) express the pit-RNA primarily from the RC transgene. Primers detecting pit-RNA expression do not distinguish expression from the transgene or endogenous locus. *Timp1* is a loading control; −RT was done without reverse transcriptase. B. DNA methylation of the endogenous (top) and transgenic (bottom) copies of the *Rasgrf1* DMD was analyzed by Sequenom MassARRAY. All mice were homozygous for a deletion of the endogenous copies of the *Rasgrf1* repeats and contained (+) or lacked (−) a paternally inherited copy of the RC transgene. Bisulfite PCR assays were specific for the endogenous (top) or RC-derived (bottom) DMD. The first 18 CpG within 210 base pairs (BPs) are shared between the two copies of the DMD; other CpGs are specific to the alleles. DNAs came from adult or embryonic somatic tissues. Robust DNA methylation and pit-RNA expression characteristic of the paternal BAC transgene recapitulates what was seen at the wild type endogenous locus. pit-RNA made by the BAC could impart methylation only at the BAC and not the endogenous locus, indicating pit-RNAs function in *cis* when transcribed from the repeat. BAC status of the fathers had no influence on methylation status at the endogenous locus in their progeny (not shown).
(TIF)
