# Supplementary figures and images for: Sequences Sufficient for Programming Imprinted Germline DNA Methylation Defined
Source: PLoS One. 2012 Mar 5;7(3):e33024. doi: 10.1371/journal.pone.0033024 (PMC3293921; doi:10.1371/journal.pone.0033024)

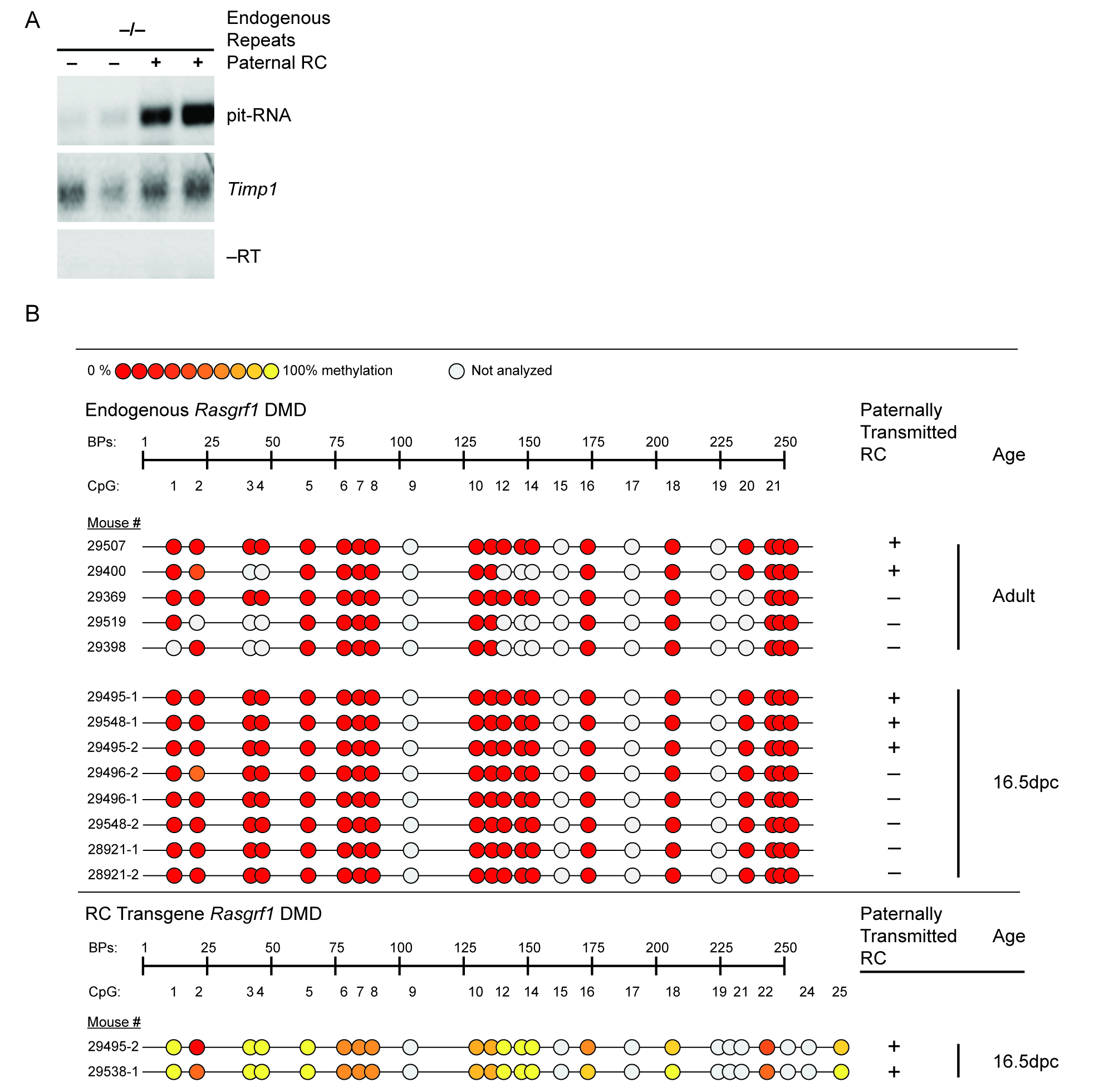

Supplement: Figure S1 — pit-RNA transcribed by the Rasgrf1 repeats controls DNA methylation only in cis and not in trans . A. Mice homozygous for a deletion of the Rasgrf1 repeats (−/−) carrying a paternally inherited RC transgene (+) express the pit-RNA primarily from the RC transgene. Primers detecting pit-RNA expression do not distinguish expression from the transgene or endogenous locus. Timp1 is a loading control; −RT was done without reverse transcriptase. B. DNA methylation of the endogenous (top) and transgenic (bottom) copies of the Rasgrf1 DMD was analyzed by Sequenom MassARRAY. All mice were homozygous for a deletion of the endogenous copies of the Rasgrf1 repeats and contained (+) or lacked (−) a paternally inherited copy of the RC transgene. Bisulfite PCR assays were specific for the endogenous (top) or RC-derived (bottom) DMD. The first 18 CpG within 210 base pairs (BPs) are shared between the two copies of the DMD; other CpGs are specific to the alleles. DNAs came from adult or embryonic somatic tissues. Robust DNA methylation and pit-RNA expression characteristic of the paternal BAC transgene recapitulates what was seen at the wild type endogenous locus. pit-RNA made by the BAC could impart methylation only at the BAC and not the endogenous locus, indicating pit-RNAs function in cis when transcribed from the repeat. BAC status of the fathers had no influence on methylation status at the endogenous locus in their progeny (not shown). (TIF) [file pone.0033024.s001.tif]
